# 2-[(*E*)-2,5-Dimethoxy­benzyl­idene]indan-1-one

**DOI:** 10.1107/S1600536809013579

**Published:** 2009-04-18

**Authors:** Abdullah Mohamed Asiri, Mehmet Akkurt, Mohie Aldin M. Zayed, Islam Ullah Khan, Muhammad Nadeem Arshad

**Affiliations:** a Chemistry Department, Faculty of Science, King Abdul-Aziz University, PO Box 80203, Jeddah 21589, Saudi Arabia; b Department of Physics, Faculty of Arts and Sciences, Erciyes University, 38039 Kayseri, Turkey; c Department of Chemistry, Government College University, Lahore, Pakistan

## Abstract

In the title compound, C_18_H_16_O_3_, the mean plane of the nine-membered indane system makes a dihedral angle of 3.71 (17)° with the benzene ring of the dimethoxy­phenyl group. The mol­ecular conformation is stabilized by intra­molecular C—H⋯O hydrogen contacts. The crystal structure is stabilized by inter­molecular C—H⋯O inter­actions, which link neighbouring mol­ecules into one-dimensional extended chains along the [100] direction. In the structure, C—H⋯π inter­actions are also observed.

## Related literature

For styryl dyes and their applications, see: Ying *et al.* (1990 ▶); He *et al.* (1995 ▶). For bond-length data, see: Allen *et al.* (1987 ▶). For details of the Flack parameter, see: Flack & Schwarzenbach (1988 ▶).
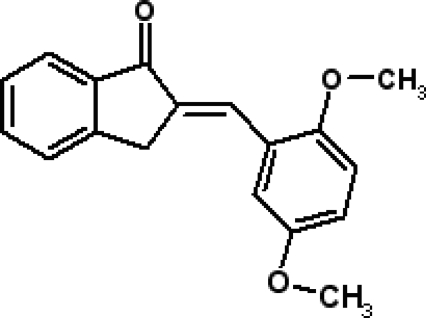


## Experimental

### 

#### Crystal data

C_18_H_16_O_3_
*M* *_r_* = 280.31
Orthorhombic, 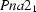

*a* = 12.925 (3) Å
*b* = 20.163 (5) Å
*c* = 5.451 (1) Å
*V* = 1420.6 (5) Å^3^
*Z* = 4
Mo *K*α radiation
μ = 0.09 mm^−1^
*T* = 296 K
0.38 × 0.09 × 0.04 mm

#### Data collection

Bruker Kappa APEXII CCD area-detector diffractometer
Absorption correction: none
8980 measured reflections
1944 independent reflections
856 reflections with *I* > 2σ(*I*)
*R*_int_ = 0.107

#### Refinement

*R*[*F* ^2^ > 2σ(*F* ^2^)] = 0.055
*wR*(*F* ^2^) = 0.115
*S* = 0.97
1944 reflections
192 parameters
1 restraint
H-atom parameters constrained
Δρ_max_ = 0.17 e Å^−3^
Δρ_min_ = −0.16 e Å^−3^

### 

Data collection: *APEX2* (Bruker, 2007 ▶); cell refinement: *APEX2*; data reduction: *SAINT* (Bruker, 2007 ▶); program(s) used to solve structure: *SIR97* (Altomare *et al.*, 1999 ▶); program(s) used to refine structure: *SHELXL97* (Sheldrick, 2008 ▶); molecular graphics: *ORTEP-3 for Windows* (Farrugia, 1997 ▶); software used to prepare material for publication: *WinGX* (Farrugia, 1999 ▶) and *PLATON* (Spek, 2009 ▶).

## Supplementary Material

Crystal structure: contains datablocks global, I. DOI: 10.1107/S1600536809013579/jh2077sup1.cif

Structure factors: contains datablocks I. DOI: 10.1107/S1600536809013579/jh2077Isup2.hkl

Additional supplementary materials:  crystallographic information; 3D view; checkCIF report

## Figures and Tables

**Table 1:** Hydrogen-bond geometry (Å, °)

*D*—H⋯*A*	*D*—H	H⋯*A*	*D*⋯*A*	*D*—H⋯*A*
C7—H7*A*⋯O1^i^	0.97	2.47	3.259 (5)	139
C10—H10⋯O1	0.93	2.52	2.891 (5)	104
C10—H10⋯O2	0.93	2.30	2.710 (5)	106
C7—H7*B*⋯*Cg*1^ii^	0.97	2.59	3.459 (4)	150
C17—H17*C*⋯*Cg*1^iii^	0.96	2.73	3.504 (4)	138

Symmetry codes: (i) 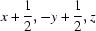; (ii) 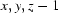; (iii) 
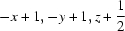
. *Cg*1 is the centroid of the C11–C16 ring.

**Fig. 1.**
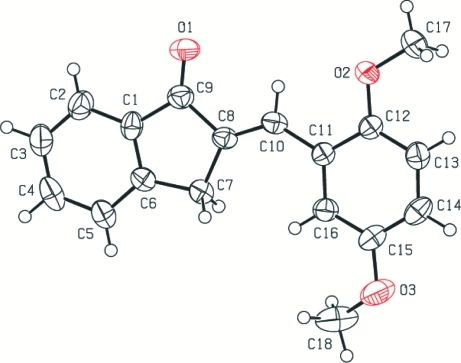
Molecular structure of (I), showing the atom labeling scheme and displacement ellipsoids are drawn at the 50% probability level. Hydrogen atoms are drawn as spheres of arbitrary radii.

**Fig. 2.**
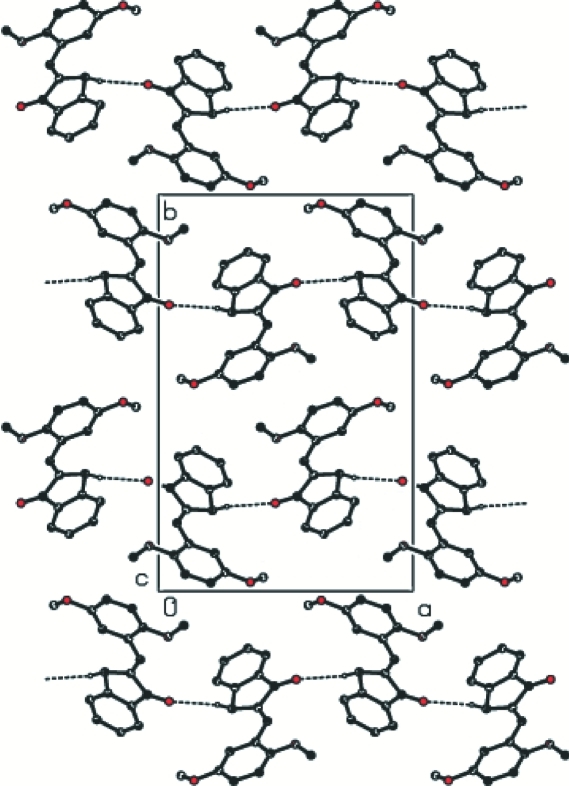
The crystal packing of the title compound, viewed down the c-axis, showing intermolecular C—H···O interactions (dashed lines). H atoms not involved in the hydrogen bonding have been omitted for clarity.

## supplementary crystallographic information

### Comment

Nonlinear optical (NLO) properties of organics have been the subject of numerous investigations in the recent years, due to their potential applications in the field of photonics. A good NLO organic material should generally contain donor and acceptor groups positioned at either ends of a conjugation path of appropriate length. The increased effective conjugation and hence, the large π-delocalization length, has been recognized as a factor leading to large third order nonlinearities. Styryl dyes are organic molecules possessing charge donor and acceptor groups, conjugated through π-electronic bridge, suitable for NLO device applications. They are widely used as optical recording medium in laser disks, laser dyes (Ying *et al.*, 1990) and optical sensitizers in various other fields (He *et al.*, 1995).

In the title compound (I) (Fig. 1), all bond lengths (Allen *et al.*, 1987) and angles are within normal ranges. The nine-membered indane ring is almost planar, with the maximum deviations of -0.017 (4) and 0.021 (4) Å for atoms C6 and C8, respectively. The mean plane of the indane ring makes a dihedral angle of 3.71 (17) °, with the benzene ring of the dimethoxy phenyl group.

The molecular conformation is stabilized by intramolecular C—H···O hydrogen contacts (Table 1). The crystal structure is stabilized by intermolecular C—H···O interactions, which link neighbouring molecules into 1-D extended chains along the [100] direction (Fig. 2). In the structure, C—H···π interactions are also observed (Table 1).

### Experimental

An equivalent molar quantities of 2,5-dimethoxybenzaldehyde (4.40 g, 26.5 mmol) and 1-indanone (3.5 g, 26.5 mmol) were dissolved in 25 ml e thanol, and then heated at reflux. Pipyridine (1 ml) was added to the solution, and reflux was continued for 5 h. The solution was cooled to room temperature, and the solid products were filtered, and washed with ethanol (25 ml) to give yellow crystals. [Yield: 5.79 g, 91%; m.p. 403 - 404 K]. IR (cm^-1^) 1689 (C=O), 1614 (C=C). ^1^H-NMR (CDCl_3_): 3.82 (3H, s, CH_3_O), 3.83 (3H, s, CH_3_O), 4.04 (2*H*, s, CH_2_), 6.83 (1*H*, d, J = 9 Hz), 6.94 (1*H*, dd, J1 = 1.8 Hz, J2 = 6 Hz), 7.23 (1*H*, d, J = 3 Hz), 7.40 (1*H*, dd, J = 7.2 Hz, J2 = 7.1 Hz), 7.54 (1*H*, d, J = 7.8 Hz), 7.6 (1*H*, d, J = 4.2 Hz), 7.9 (1*H*, d, J = 9 Hz), 8.1 (1*H*, s, CH=C).

### Refinement

All H atoms bonded to the C atoms were positioned geometrically, with C—H distances in the range 0.93–0.97 Å, and refined using a riding approximation model, with *U*_iso_(H) = 1.5*U*_eq_ of the carrier atom for methyl H and 1.2*U*_eq_ for the remaining H atoms. The calculation of the Flack (Flack & Schwarzenbach, 1988) parameter was suppressed by the use of the MERG 4 instruction in *SHELXL*97 (Sheldrick, 2008), as the lack of anomalous scatterers did not allow the determination of the absolute configuration from the X-ray measurements.

### Crystal data

**Table:**

C_18_H_16_O_3_	*F*(000) = 592
*M**_r_* = 280.31	*D*_x_ = 1.311 Mg m^−^^3^
Orthorhombic, *P**n**a*2_1_	Mo *K*α radiation, λ = 0.71073 Å
Hall symbol: P 2c -2n	Cell parameters from 598 reflections
*a* = 12.925 (3) Å	θ = 2.6–18.0°
*b* = 20.163 (5) Å	µ = 0.09 mm^−^^1^
*c* = 5.451 (1) Å	*T* = 296 K
*V* = 1420.6 (5) Å^3^	Prism, yellow
*Z* = 4	0.38 × 0.09 × 0.04 mm

### Data collection

**Table:**

Bruker Kappa APEXII CCD area-detector diffractometer	856 reflections with *I* > 2σ(*I*)
Radiation source: sealed tube	*R*_int_ = 0.107
graphite	θ_max_ = 28.3°, θ_min_ = 2.6°
φ and ω scans	*h* = −16→17
8980 measured reflections	*k* = −26→25
1944 independent reflections	*l* = −7→4

### Refinement

**Table:**

Refinement on *F*^2^	Primary atom site location: structure-invariant direct methods
Least-squares matrix: full	Secondary atom site location: difference Fourier map
*R*[*F*^2^ > 2σ(*F*^2^)] = 0.055	Hydrogen site location: inferred from neighbouring sites
*wR*(*F*^2^) = 0.115	H-atom parameters constrained
*S* = 0.97	*w* = 1/[σ^2^(*F*_o_^2^) + (0.037*P*)^2^] where *P* = (*F*_o_^2^ + 2*F*_c_^2^)/3
1944 reflections	(Δ/σ)_max_ < 0.001
192 parameters	Δρ_max_ = 0.17 e Å^−^^3^
1 restraint	Δρ_min_ = −0.16 e Å^−^^3^

### Special details

**Table:**

Geometry. Bond distances, angles *etc*. have been calculated using the rounded fractional coordinates. All su's are estimated from the variances of the (full) variance-covariance matrix. The cell e.s.d.'s are taken into account in the estimation of distances, angles and torsion angles
Refinement. Refinement on *F*^2^ for ALL reflections except those flagged by the user for potential systematic errors. Weighted *R*-factors *wR* and all goodnesses of fit *S* are based on *F*^2^, conventional *R*-factors *R* are based on *F*, with *F* set to zero for negative *F*^2^. The observed criterion of *F*^2^ > σ(*F*^2^) is used only for calculating -*R*-factor-obs *etc*. and is not relevant to the choice of reflections for refinement. *R*-factors based on *F*^2^ are statistically about twice as large as those based on *F*, and *R*-factors based on ALL data will be even larger.

### Fractional atomic coordinates and isotropic or equivalent isotropic displacement parameters (Å^2^)

**Table:**

	*x*	*y*	*z*	*U*_iso_*/*U*_eq_	
O1	0.4588 (2)	0.22227 (16)	0.5837 (6)	0.0619 (13)	
O2	0.4627 (2)	0.38576 (15)	1.1861 (5)	0.0507 (11)	
O3	0.8608 (2)	0.47706 (17)	1.0485 (6)	0.0683 (16)	
C1	0.6059 (3)	0.2111 (2)	0.3166 (8)	0.0400 (16)	
C2	0.5790 (3)	0.1610 (2)	0.1549 (9)	0.0507 (17)	
C3	0.6488 (3)	0.1442 (2)	−0.0268 (9)	0.0533 (17)	
C4	0.7428 (4)	0.1762 (2)	−0.0452 (8)	0.0553 (19)	
C5	0.7699 (3)	0.2264 (2)	0.1200 (8)	0.0460 (17)	
C6	0.6999 (3)	0.2433 (2)	0.3014 (7)	0.0360 (14)	
C7	0.7111 (3)	0.2962 (2)	0.4948 (7)	0.0347 (14)	
C8	0.6098 (3)	0.2932 (2)	0.6309 (7)	0.0350 (14)	
C9	0.5446 (3)	0.2392 (2)	0.5186 (7)	0.0423 (16)	
C10	0.5726 (3)	0.3277 (2)	0.8213 (8)	0.0357 (14)	
C11	0.6191 (3)	0.3804 (2)	0.9655 (7)	0.0327 (14)	
C12	0.5612 (3)	0.4089 (2)	1.1572 (7)	0.0353 (14)	
C13	0.6046 (3)	0.4569 (2)	1.3073 (8)	0.0423 (17)	
C14	0.7049 (3)	0.4787 (2)	1.2631 (8)	0.0450 (17)	
C15	0.7623 (3)	0.4521 (2)	1.0747 (9)	0.0430 (17)	
C16	0.7202 (3)	0.4039 (2)	0.9271 (7)	0.0393 (16)	
C17	0.4010 (3)	0.4138 (2)	1.3758 (8)	0.0570 (19)	
C18	0.9112 (3)	0.4649 (3)	0.8234 (10)	0.072 (2)	
H2	0.51570	0.13930	0.16830	0.0600*	
H3	0.63220	0.11080	−0.13810	0.0640*	
H4	0.78880	0.16430	−0.16890	0.0670*	
H5	0.83340	0.24790	0.10800	0.0550*	
H7A	0.76870	0.28660	0.60320	0.0410*	
H7B	0.72130	0.33940	0.42110	0.0410*	
H10	0.50600	0.31600	0.86900	0.0430*	
H13	0.56670	0.47460	1.43690	0.0500*	
H14	0.73350	0.51150	1.36190	0.0540*	
H16	0.75940	0.38640	0.79940	0.0470*	
H17A	0.43230	0.40480	1.53210	0.0860*	
H17B	0.33300	0.39470	1.37100	0.0860*	
H17C	0.39610	0.46090	1.35240	0.0860*	
H18A	0.92540	0.41830	0.80830	0.1070*	
H18B	0.97500	0.48920	0.81820	0.1070*	
H18C	0.86750	0.47880	0.69060	0.1070*	

### Atomic displacement parameters (Å^2^)

**Table:**

	*U*^11^	*U*^22^	*U*^33^	*U*^12^	*U*^13^	*U*^23^
O1	0.0348 (17)	0.084 (3)	0.067 (2)	−0.0157 (19)	0.0072 (17)	−0.021 (2)
O2	0.0369 (17)	0.064 (2)	0.0512 (19)	−0.0076 (17)	0.0123 (17)	−0.0217 (17)
O3	0.055 (2)	0.084 (3)	0.066 (3)	−0.0298 (19)	−0.003 (2)	−0.007 (2)
C1	0.050 (3)	0.038 (3)	0.032 (2)	0.004 (2)	−0.007 (3)	−0.005 (3)
C2	0.054 (3)	0.051 (3)	0.047 (3)	−0.001 (2)	−0.010 (3)	−0.010 (3)
C3	0.062 (3)	0.048 (3)	0.050 (3)	0.009 (3)	−0.003 (3)	−0.014 (3)
C4	0.063 (3)	0.059 (4)	0.044 (3)	0.027 (3)	0.005 (3)	−0.006 (3)
C5	0.047 (3)	0.051 (3)	0.040 (3)	0.011 (2)	0.002 (2)	−0.005 (3)
C6	0.034 (2)	0.041 (3)	0.033 (2)	0.005 (2)	−0.001 (2)	0.004 (2)
C7	0.036 (2)	0.044 (3)	0.024 (2)	0.003 (2)	−0.0019 (19)	0.003 (2)
C8	0.029 (2)	0.042 (3)	0.034 (2)	0.001 (2)	−0.002 (2)	0.002 (2)
C9	0.035 (2)	0.052 (3)	0.040 (3)	−0.002 (2)	−0.002 (2)	−0.003 (2)
C10	0.030 (2)	0.050 (3)	0.027 (2)	0.000 (2)	0.000 (2)	0.003 (2)
C11	0.034 (2)	0.036 (3)	0.028 (2)	0.001 (2)	−0.004 (2)	0.001 (2)
C12	0.037 (2)	0.038 (3)	0.031 (2)	−0.001 (2)	−0.004 (2)	0.001 (2)
C13	0.045 (3)	0.043 (3)	0.039 (3)	0.007 (2)	−0.006 (3)	−0.004 (3)
C14	0.052 (3)	0.041 (3)	0.042 (3)	−0.007 (2)	−0.020 (3)	0.005 (2)
C15	0.046 (3)	0.043 (3)	0.040 (3)	−0.013 (2)	−0.007 (2)	0.003 (3)
C16	0.035 (2)	0.046 (3)	0.037 (3)	−0.003 (2)	0.001 (2)	−0.001 (2)
C17	0.053 (3)	0.069 (4)	0.049 (3)	0.000 (3)	0.016 (2)	−0.014 (3)
C18	0.048 (3)	0.104 (5)	0.063 (4)	−0.023 (3)	−0.009 (3)	0.027 (4)

### Geometric parameters (Å, °)

**Table:**

O1—C9	1.213 (5)	C13—C14	1.390 (6)
O2—C12	1.365 (5)	C14—C15	1.376 (6)
O2—C17	1.423 (5)	C15—C16	1.374 (6)
O3—C15	1.376 (5)	C2—H2	0.9300
O3—C18	1.411 (6)	C3—H3	0.9300
C1—C2	1.385 (6)	C4—H4	0.9300
C1—C6	1.380 (6)	C5—H5	0.9300
C1—C9	1.470 (6)	C7—H7A	0.9700
C2—C3	1.382 (6)	C7—H7B	0.9700
C3—C4	1.379 (6)	C10—H10	0.9300
C4—C5	1.399 (6)	C13—H13	0.9300
C5—C6	1.383 (6)	C14—H14	0.9300
C6—C7	1.507 (6)	C16—H16	0.9300
C7—C8	1.506 (5)	C17—H17A	0.9600
C8—C9	1.507 (6)	C17—H17B	0.9600
C8—C10	1.339 (6)	C17—H17C	0.9600
C10—C11	1.452 (6)	C18—H18A	0.9600
C11—C12	1.408 (5)	C18—H18B	0.9600
C11—C16	1.406 (6)	C18—H18C	0.9600
C12—C13	1.386 (6)		
			
O1···C7^i^	3.259 (5)	C14···H17C^viii^	2.8600
O2···C5^ii^	3.384 (5)	C15···H17C^viii^	2.9600
O2···C4^ii^	3.351 (6)	C15···H7B^vii^	3.0000
O1···H10	2.5200	C16···H18C	2.7500
O1···H2	2.9100	C16···H7B	3.0500
O1···H7A^i^	2.4700	C16···H7A	3.0200
O2···H10	2.3000	C16···H7B^vii^	2.9900
O3···H18B^iii^	2.6700	C16···H18A	2.7500
C3···C8^iv^	3.572 (6)	C17···H13	2.4900
C3···C9^iv^	3.409 (6)	C18···H16	2.5200
C4···C7^iv^	3.508 (6)	H2···O1	2.9100
C4···C8^iv^	3.411 (6)	H2···H18A^x^	2.5600
C4···O2^v^	3.351 (6)	H7A···C4^vii^	2.9600
C4···C17^v^	3.569 (6)	H7A···C5^vii^	3.0700
C5···C17^v^	3.579 (6)	H7A···C16	3.0200
C5···O2^v^	3.384 (5)	H7A···H16	2.2800
C6···C11^iv^	3.476 (6)	H7A···O1^vi^	2.4700
C6···C10^iv^	3.529 (6)	H7B···C11^iv^	2.9300
C7···C11^iv^	3.552 (6)	H7B···C12^iv^	2.8800
C7···O1^vi^	3.259 (5)	H7B···C13^iv^	2.8800
C7···C16	3.207 (6)	H7B···C14^iv^	2.9500
C7···C4^vii^	3.508 (6)	H7B···C15^iv^	3.0000
C7···C12^iv^	3.508 (6)	H7B···C16^iv^	2.9900
C8···C12^iv^	3.536 (6)	H7B···C16	3.0500
C8···C4^vii^	3.411 (6)	H7B···H16	2.3200
C8···C3^vii^	3.572 (6)	H10···O1	2.5200
C9···C3^vii^	3.409 (6)	H10···O2	2.3000
C10···C6^vii^	3.529 (6)	H13···C17	2.4900
C11···C7^vii^	3.552 (6)	H13···H17A	2.3000
C11···C6^vii^	3.476 (6)	H13···H17C	2.2700
C12···C8^vii^	3.536 (6)	H14···H18C^vii^	2.5800
C12···C7^vii^	3.508 (6)	H16···C7	2.5400
C13···C17^viii^	3.512 (6)	H16···C8	2.8500
C14···C17^viii^	3.321 (6)	H16···C18	2.5200
C15···C17^viii^	3.597 (6)	H16···H7A	2.2800
C16···C7	3.207 (6)	H16···H7B	2.3200
C17···C5^ii^	3.579 (6)	H16···H18A	2.2400
C17···C15^ix^	3.597 (6)	H16···H18C	2.4000
C17···C4^ii^	3.569 (6)	H17A···C10^vii^	2.8600
C17···C13^ix^	3.512 (6)	H17A···C13	2.7500
C17···C14^ix^	3.321 (6)	H17A···H13	2.3000
C4···H7A^iv^	2.9600	H17B···C4^ii^	2.9200
C4···H17B^v^	2.9200	H17B···C5^ii^	2.9200
C5···H7A^iv^	3.0700	H17C···C13	2.7100
C5···H17B^v^	2.9200	H17C···H13	2.2700
C7···H16	2.5400	H17C···C13^ix^	2.9800
C8···H16	2.8500	H17C···C14^ix^	2.8600
C10···H17A^iv^	2.8600	H17C···C15^ix^	2.9600
C11···H7B^vii^	2.9300	H18A···C16	2.7500
C12···H7B^vii^	2.8800	H18A···H16	2.2400
C13···H17A	2.7500	H18A···H2^xi^	2.5600
C13···H7B^vii^	2.8800	H18B···O3^xii^	2.6700
C13···H17C	2.7100	H18C···C16	2.7500
C13···H17C^viii^	2.9800	H18C···H14^iv^	2.5800
C14···H7B^vii^	2.9500	H18C···H16	2.4000
			
C12—O2—C17	118.1 (3)	C3—C2—H2	121.00
C15—O3—C18	117.0 (4)	C2—C3—H3	120.00
C2—C1—C6	121.7 (4)	C4—C3—H3	120.00
C2—C1—C9	128.5 (4)	C3—C4—H4	120.00
C6—C1—C9	109.8 (4)	C5—C4—H4	120.00
C1—C2—C3	118.1 (4)	C4—C5—H5	121.00
C2—C3—C4	120.8 (4)	C6—C5—H5	121.00
C3—C4—C5	120.8 (4)	C6—C7—H7A	111.00
C4—C5—C6	118.3 (4)	C6—C7—H7B	111.00
C1—C6—C5	120.2 (4)	C8—C7—H7A	111.00
C1—C6—C7	112.1 (3)	C8—C7—H7B	111.00
C5—C6—C7	127.7 (4)	H7A—C7—H7B	109.00
C6—C7—C8	103.5 (3)	C8—C10—H10	115.00
C7—C8—C9	108.4 (3)	C11—C10—H10	115.00
C7—C8—C10	132.3 (4)	C12—C13—H13	120.00
C9—C8—C10	119.3 (4)	C14—C13—H13	120.00
O1—C9—C1	127.1 (4)	C13—C14—H14	120.00
O1—C9—C8	126.6 (4)	C15—C14—H14	120.00
C1—C9—C8	106.3 (3)	C11—C16—H16	119.00
C8—C10—C11	130.6 (4)	C15—C16—H16	119.00
C10—C11—C12	118.7 (4)	O2—C17—H17A	109.00
C10—C11—C16	123.4 (4)	O2—C17—H17B	110.00
C12—C11—C16	117.8 (4)	O2—C17—H17C	109.00
O2—C12—C11	116.2 (3)	H17A—C17—H17B	110.00
O2—C12—C13	123.2 (3)	H17A—C17—H17C	109.00
C11—C12—C13	120.5 (4)	H17B—C17—H17C	109.00
C12—C13—C14	119.7 (4)	O3—C18—H18A	109.00
C13—C14—C15	120.6 (4)	O3—C18—H18B	109.00
O3—C15—C14	115.7 (4)	O3—C18—H18C	109.00
O3—C15—C16	124.4 (4)	H18A—C18—H18B	109.00
C14—C15—C16	120.0 (4)	H18A—C18—H18C	109.00
C11—C16—C15	121.3 (4)	H18B—C18—H18C	110.00
C1—C2—H2	121.00		
			
C17—O2—C12—C11	−179.2 (3)	C6—C7—C8—C10	−179.9 (4)
C17—O2—C12—C13	1.8 (6)	C7—C8—C9—O1	179.0 (4)
C18—O3—C15—C14	−163.3 (4)	C7—C8—C9—C1	−0.7 (4)
C18—O3—C15—C16	16.5 (6)	C10—C8—C9—O1	−0.4 (6)
C6—C1—C2—C3	−0.8 (6)	C10—C8—C9—C1	180.0 (4)
C9—C1—C2—C3	178.0 (4)	C7—C8—C10—C11	−0.8 (8)
C2—C1—C6—C5	0.7 (6)	C9—C8—C10—C11	178.4 (4)
C2—C1—C6—C7	179.4 (4)	C8—C10—C11—C12	179.3 (4)
C9—C1—C6—C5	−178.3 (4)	C8—C10—C11—C16	−1.9 (7)
C9—C1—C6—C7	0.5 (5)	C10—C11—C12—O2	−2.4 (5)
C2—C1—C9—O1	1.6 (7)	C10—C11—C12—C13	176.6 (4)
C2—C1—C9—C8	−178.8 (4)	C16—C11—C12—O2	178.8 (3)
C6—C1—C9—O1	−179.5 (4)	C16—C11—C12—C13	−2.2 (6)
C6—C1—C9—C8	0.1 (5)	C10—C11—C16—C15	−177.4 (4)
C1—C2—C3—C4	0.4 (6)	C12—C11—C16—C15	1.4 (6)
C2—C3—C4—C5	0.1 (7)	O2—C12—C13—C14	−178.9 (4)
C3—C4—C5—C6	−0.3 (6)	C11—C12—C13—C14	2.2 (6)
C4—C5—C6—C1	−0.1 (6)	C12—C13—C14—C15	−1.3 (6)
C4—C5—C6—C7	−178.7 (4)	C13—C14—C15—O3	−179.6 (4)
C1—C6—C7—C8	−0.9 (4)	C13—C14—C15—C16	0.5 (6)
C5—C6—C7—C8	177.8 (4)	O3—C15—C16—C11	179.6 (4)
C6—C7—C8—C9	0.9 (4)	C14—C15—C16—C11	−0.6 (6)

Symmetry codes: (i) *x*−1/2, −*y*+1/2, *z*; (ii) *x*−1/2, −*y*+1/2, *z*+1; (iii) −*x*+2, −*y*+1, *z*+1/2; (iv) *x*, *y*, *z*−1; (v) *x*+1/2, −*y*+1/2, *z*−1; (vi) *x*+1/2, −*y*+1/2, *z*; (vii) *x*, *y*, *z*+1; (viii) −*x*+1, −*y*+1, *z*−1/2; (ix) −*x*+1, −*y*+1, *z*+1/2; (x) *x*−1/2, −*y*+1/2, *z*−1; (xi) *x*+1/2, −*y*+1/2, *z*+1; (xii) −*x*+2, −*y*+1, *z*−1/2.

### Hydrogen-bond geometry (Å, °)

**Table:**

*D*—H···*A*	*D*—H	H···*A*	*D*···*A*	*D*—H···*A*
C7—H7A···O1^vi^	0.97	2.47	3.259 (5)	139
C10—H10···O1	0.93	2.52	2.891 (5)	104
C10—H10···O2	0.93	2.30	2.710 (5)	106
C7—H7B···Cg1^iv^	0.97	2.59	3.459 (4)	150
C17—H17C···Cg1^ix^	0.96	2.73	3.504 (4)	138

Symmetry codes: (vi) *x*+1/2, −*y*+1/2, *z*; (iv) *x*, *y*, *z*−1; (ix) −*x*+1, −*y*+1, *z*+1/2.
